# Clinical Society of Maryland

**Published:** 1896-03

**Authors:** H. O. Reik

**Affiliations:** Secretary; 525 North Howard Street; Baltimore


### Society Reports.

### CLINICAL SOCIETY OF MARYLAND.

Baltimore. December 20, 1896.

The 315th regular meeting of the Clinical Society of Maryland was called to order by the president, Dr. J. M. Hundley.

Dr. L. McLane Tiffany read a paper on

### GUNSHOT WOUNDS OF THE PERITONEAL CAVITY AND CONTAINED VISCERA.

Discussion.

Dr. Randolph Winslow: I am on the card to open this discussion, and will simply report the cases I have seen in the University of Maryland since 1888. During the last three years of my service there, we have had four cases of gunshot wounds of the abdomen. Three were operated upon by me and one by Dr. Spruill. The first case upon which I operated has been previously reported to this society, and is as follows:

Case I.—Man, age 60. On the 21st of December, 1893, about 6:30 p. m., while stealing coal, was shot by a watchman and received a number of wounds of the small intestine. He was brought to the hospital about 1:30 a. m. the next day, and I saw him at noon. There was no evidence of shock, and his condition was pretty good. There was a small, blackened area about the external wound, which was situated in the right flank. His temperature was 101; pulse, 104. He was anæsthetized, properly prepared for operation and on exploration, the wound was found to lead into the peritoneal cavity. The abdomen was opened and flushed out with hot water. Four wounds were found in the bowel and some fæcal matter in the peritoneum. The intestines were washed, sewed up, and put back, and the abdominal wound packed with gauze, because, owing to the free oozing, it was thought best not to close it. The bullet could be felt under the skin, near the crest of the illium, having passed entirely through the bone. The patient was somewhat shocked, but rallied promptly and eventually recovered.

CASE II.—This case occurred last year, while I was in Cumberland, attending the meeting of the State Faculty. The man who had been shot about twenty-four hours previously, was in a septic condition and had general peritonitis when brought into the hospital. Dr. Spruill promptly opened the abdomen and sewed up the wounds which were nine in number, but the patient did not rally, and soon died.

CASE III.—White man, aged 58. On July 4, while crossing a vacant lot, some boys fired a cannon filled with big shot, and the man was hit, though, at the time, he did not feel it. The shot struck him in the left side below the apex of the heart. He walked home and to the hospital. Dr. Spruill examined him and on introducing a probe, found that the bullet had entered the peritoneal cavity. He had vomited some blood clots. An incision was made in the left hypogastric region, and the stomach exposed. The bullet was found to have passed through the wall of the stomach and the wound was closed by a plug of mucous membrane. No extravasation had occurred. The hole was closed by interrupted sutures. After the operation, he vomited blood clots several times. His temperature was 101½; pulse, 88. He developed a bronchitis on the fourth day. The external wound had suppurated, but the peritoneum was not invaded, and the patient got well.

CASE IV.—This was not so fortunate. On the 7th of September, this man was shot, and brought to the hospital within an hour. The bullet had entered the abdominal cavity and besides five wounds of the small intestine, there was a number of the mesentery. Laparotomy was performed, the bleeding vessels ligated, perforations closed, the cavity flushed with sterilized water, and the incision closed. The operation was performed at midnight; the patient was collapsed and in such a condition that it was feared he would die on the table, consequently, as careful a search as was desired, could not be made. The wounds that were formed were supposed to be all that were present, but that proved to be a mistake. On September 8, the patient was doing well. September 9, some pain in the lower abdomen. September 10, vomited frequently. Gave enema to cause free movement of the bowel and administered strychnia hypodermically. September 11, five movements, upper abdomen distended, and vomited frequently. September 12, seemed better. Had not vomited for twelve hours. Taking milk and lime water and calomel every two hours. Died that night. Autopsy showed a hole in the cæcum. The wounds that were sutured had held firmly. Unfortunately, one hole had been overlooked.

Case I.—My attention was first called practically to penetrating wounds of the abdomen in July, 1881. On the 4th of July of that year, a colored man was admitted to the University Hospital, with a pistol wound, the ball entering the left side of the abdomen at a point 1½ inches from the linea alba and 3 inches from the umbilicus, making a circular hole with clean-cut edge about the size of the end of the little finger, through which about an inch of omentum protruded. There was no hæmorrhage, and but little pain, the temperature was normal, and the pulse 80, and full and strong. As I could not replace the omentum, it was ligatured and the redundant portion cut off. The wound was slightly enlarged and a drainage tube introduced. Two ice bladders were applied to the abdomen, and a grain of opium administered every three hours. On the third day, his temperature rose from 99, in the morning, to 101 3-5 in the evening; the pulse from 80 to 126; and the respiration to 32; his abdomen became tympanitic, but there was very little tenderness on pressure. The onset of peritonitis was feared, but the next morning, the temperature dropped to 99 4-5 and the pulse to 104; and subsequently no alarming symtoms occurred, and the man was discharged from the hospital in 30 days, entirely well. This case came under my care just previous to the publication of Dr. J. Marion Sim's famous article advocating laparotomy in this class of injuries, and the favorable result is to be attributed to good fortune, rather than to good treatment. It is evident that the ball spent itself in penetrating the abdominal wall and did not wound the intestines.

Case II.—Pistol wound of epigastrium. June 3, 1894. S. P., negress, was shot with a large revolver, the bullet entering the epigastrium, one and one-half inches from the linea alba, and three inches below the ensiform cartilage. From the situation of the wound, it was thought that the stomach, or large intestine, was injured, but there was no vomiting of blood nor bloody stools. There was some pain, but no elevation of the temperature, and she was let alone. She lived five days and two hours, and died with symptoms suggestive of peritonitis. At the autopsy, no peritonitis was found, nor any wound of the abdominal viscera. Some fluid was found in the peritoneal cavity, and the peritoneum detached, with an enormous blood clot under it. A long slit was found in the aorta below the cœliac axis and a counter opening opposite the third lumbar vertebra. There were thus two large openings in the aorta, and yet she lived five days and two hours.

CASE III.—Pistol wounds of small intestines. Twenty-seven holes in intestines and bladder; death in eighteen hours. C. W., admitted on same day as above, was shot in the hypogastric region, over the pubes, with a large pistol. One wound was in the hypogastrium and a bullet was cut out from the integument one inch distant. Another wound was found upon the buttock, near the anus and a bullet was removed from the skin three inches distant. Was admitted to the hospital during the night. Pulse good and shock not marked. Urine bloody. When seen by me the next day, was in collapse; pulseless, vomiting, and in great pain. Autopsy: The bullets were found to have pursued nearly parallel courses, one entering in front and lodging under the skin of the buttock; the other entering behind and lodging beneath the skin of the abdomen. The peritoneal cavity was filled with blood, fæces, pepper pods, and cherry-stones; the lower portion of the small intestine, for about six feet was riddled, there being twenty-five holes in the bowel, one in the mesentery, one in the top of the bladder, and one in the base; altogether, twenty-eight wounds was the result of these two bullets.

Laparotomy should have been performed in both of these cases, though there is scarcely a chance that a favorable result could have been obtained. I only saw the last case when he was in collapse.

CASE IV.—Pistol wound of liver; death in eighteen or twenty hours. D. G., colored, was shot on the same day as the two preceding cases. The wound was on the right side, in the anterior axillary line, about two inches from the nipple in the fifth intercostal space, and the ball ranging downwards, fractured the sixth rib and entered the abdomen between the sixth and seventh ribs. Much bleeding and marked shock followed. Local pain and pain in the right shoulder. There was no escape of bile from the wound. The patient vomited the contents of the stomach, but no blood. A wound of the liver was diagnosticated. The patient never rallied from the shock, and died in the evening of the same day. Autopsy: The bullet was found to have passed through the upper surface of the liver and to be embedded in the substance of the diaphragm.

CASE V.—Pistol wound of back, perforation of the small intestine, death in twenty-nine hours, from peritonitis. W. G., colored, was shot while running away from a policeman, the bullet entering his back one inch to the left of the second lumbar vertebra, and a probe could not be made to follow its track. Seven hours after the injury, the pulse was 82; respiration, 32; temperature, 98.8; and there was no shock. Soon pain about the umbilicus set in, and rigidity of the abdominal muscles, vomiting and a bloody alvine dejection, and there was numbness of the parts supplied by the left anterior crural nerve. The temperature began to rise and in twelve hours reached 100.2; pulse, 90; respiration, 48, and thoracic in character, the abdominal tenderness remaining. A consultation was held in regard to the propriety of performing laparotomy in this case, but the consultant was opposed to it. The patient died of peritonitis in twenty-nine hours. Autopsy: The ball entered the back opposite the second lumbar vertebra, grazed the third lumbar vertebra, then passed into the peritoneal cavity, pierced the small intestine in two places, and finally dropped into the pelvis. Fæces had escaped and an intense general peritonitis was set up. Laparotomy ought to have been performed in this case.

CASE VI.—Gunshot wound, supposed to have penetrated the abdominal cavity. Operation declined. Recovery. Michael Kane, age 45, admitted December 2, 1893. He was shot a short time before admission, with a pistol, the bullet entering three inches to the left of the sternum and one inch below, just over the left costal margin. Dr. Spruill examined him under an anæsthetic and thought that the ball had gone into the peritoneal cavity. After examining the man, I recommended an exploratory laparotomy, which he declined. No serious symptoms supervened and he left the hospital on December 20. The highest temperature was 101, and pulse, 80.

After a consideration of these cases, I agree with Dr. Tiffany that a person who comes in contact with a case of penetrating wound of the abdomen has not done his duty if he fails to give the patient an opportunity for life by performing section. A certain number of cases will recover without operation, but such treatment is simply working in the dark, and in those cases, it is probable that the viscera have not been injured. If they have been injured, it is almost inevitable that death will follow. No surgical rule is better established than that such wounds should be treated by an exploring section, and if necessary by the sewing up of the wounds and ligation of vessels that have been ruptured. There are many points in regard to the technique of such operations which I will not now take up your time in considering, but I wish again to express my opinion in thorough conformity with what has been said, that a surgeon is not justified in permitting a patient to go without laparotomy who has received a penetrating wound of the abdomen. There is but one thing to do in these cases and that is to perform laparotomy and be further guided by the circumstances of the case.

DR. J. W. CHAMBERS: I have been very much interested in the relation of these cases, and think the position taken is the proper one; that every case of penetratng wound of the abdomen demands a laparotomy. One important point in the consideration of these cases is, that no one can draw any deduction whatever from the amount of shock present. There is no relation between the shock and the injury. I have seen recently a man with perforating wound, walking into the hospital with but slight assistance. Another strong point in favor of the operation is, that you do not know anything about what has been injured until you open the abdomen. While there is a rule ordinarily accepted that a bullet entering this cavity and traveling from side to side makes many wounds, while one from before back will make but few, it cannot be relied upon at all, as is shown in one of the cases related by Dr. Winslow. Only occasionally does a patient recover without operative interference and the operation should be done early. It will be a long time before we can draw conclusions from the cases reported as they vary so in character. Within the last six months, I have operated upon three cases, and all recovered; but, previously to that, I had operated upon four, and all died. All of my last three cases were operated upon immediately after the injury, while the others were not seen early enough.

DR. B. B. BROWN: Some of us remember the paper read by Dr. J. Marion Sims. He was the first to advise laparotomy in these cases, and I was especially impressed by Dr. Tiffany's resume, which varies but little from the rules laid down by Sims, I think in 1881, some fourteen years ago. This mode of treatment was at first severely criticised; now, however, the best surgeons recognize it as perfectly correct, and it shows one more important truth brought forth by that great man.

DR. H. H. BIEDLER: I have had some little experience in these cases, and recently have had several unfortunate ones. In one, a colored boy, who had been shot by one of his companions, an extensive laparotomy was performed, the abdomen being opened, and the incision, which commenced at the sternum, ended at the pubes. Only one wound was discovered in the viscera and that was closed, the bullet not found. The boy died, and at the autopsy the bullet was found beneath the right nipple. The question arises whether laparotomy had not more do with the determination of that life than had the injury. I have seen a large number of cases where this operation was done and followed by decided benefit. Another case I saw, was that of a man brought into the Baltimore University with a gunshot injury, which I took to be a penetrating wound of the abdominal cavity. He presented evidences of shock, and, by the way, I am not inclined to agree with what has been said to the effect that shock does not play any part in the estimation of what shall be done with the case, but, on the other hand, am inclined to think that if the viscera are injured, the patient will have a decided amount of shock. This patient died within a very short time, and, at the autopsy, he was found to have a penetrating ulcer of the stomach and a general peritonitis.

I think, as all have said, it is wise to make an exploratory incision wherever you have such injuries to deal with; that has been the tenor of the best surgeons for many years. As to the results, and whether it is best to do it in a given number of cases, no one can tell.

DR. J. M. T. FINNEY: I am sorry I was too late to hear Dr. Tiffany's papers, but his opinions, as I get them from the discussion, are in accord with the best surgeons of the day. Since the day when Dr. ———————'s paper appeared in 1887, in which he reviewed the literature of the subject, there has been very little diversity of opinion as to the advisability of this method of treatment. I subscribe to it most heartily.

DR. J. G. JAY: I agree with the practice generally pursued, as stated by those who have spoken before me, and consider it right to follow this line of treatment.

DR. L. MCLANE TIFFANY: The only point I would speak of is the question of shock. In my paper, I quoted Miles in reference to that shock which occurs with the injury. If the shock is progressive, it means internal hæmorrhage. When the patient is first seen he may be profoundly shocked and not be much disturbed, but if he continues to become more shocked it means hæmorrhage, and there is all the more demand for operation. Shock at the time of the injury does not mean hæmorrhage, but later on, it does.

DR. RANDOLPH WINSLOW reported a case of rupture of the liver—laparotomy—death. H. E., white, age 25, admitted to the Maryland University Hospital, on September 30, 1895. Whilst riding on the shaft of a loaded cart, about 8:30 A. M., he attempted to get off, and his foot becoming entangled in the reins, he fell and the wheel passed over his abdomen obliquely, from the right hypochondrium, to the left iliac crest. He was brought into the hospital at once, greatly shocked, with pain in the abdomen, quick and weak pulse, and temperature of 95 degrees. I saw him at eleven A. M. Face blanched; temperature, 96; pulse, fairly good; some pain in the abdomen; some bruises over the right side, but none on the abdomen. There was marked percussion dullness over the lower portion of the belly. A rupture of some of the abdominal viscera, probably the liver, with hæmorrhage; was diagnosticated, and laparotomy was advised. The operation was performed at 1 P. M. An incision was made in the linea alba from the ensiform cartilage to the pubes, and even before the peritoneum was opened, the dark color of the tissues indicated the presence of blood in the peritoneal cavity which gushed out to the amount of a quart or more, when the peritoneum was incised. The intestines were examined and found to be uninjured but the liver was extensively lacerated on its posterior and inferior aspect in two places. The belly was thoroughly washed out and the lacerations of the liver packed with gauze. The patient stood the operation well and seemed to rally somewhat afterwards, but died on the same night about twelve hours after the operation. At the autopsy, the peritoneal cavity was found to be filled with blood showing that the packing had not prevented hæmorrhage. I consider laparotomy to be urgently demanded in this class of cases, though the outlook for a successful result is very poor.

DISCUSSION.

DR. J. W. CHAMBERS: I would report a case of this character, which occurred to me some six months ago. A young man racing across the street with a friend fell, and it was supposed struck his right side on the curb. His friend thought that he was not seriously injured and did not call for medical assistance. He was carried into a store and was kept all night. He rallied toward morning and was then carried home. When I saw him, his abdomen was distended but no dullness could be made out. An operation was suggested to the family but refused, and he died twenty-one hours after the fall. At the autopsy, it was shown that the liver was practically torn to pieces. It was crushed, and one part was hanging way down, but not absolutely separated. The gall bladder was not ruptured. There was an immense amount of blood in the cavity.

In another similar case, a gentleman was crossing the street ahead of a cab, and it is supposed was struck by the pole though those who saw the accident were not sure that he was struck. I was at the hospital when he came in. He was dead when he arrived. At the autopsy, most of the liver was found in the pelvis. Scarcely half an ounce of fluid in the cavity. His heart was probably stopped when he was struck and he died at once. There was no appearance of injury on the surface, and no hæmorrhage.

DR. S. K. MERRICK exhibited a "Poly-aural Stethoscope" of his own invention and explained its use. It is designed for use in teaching, and will accommodate five students at a time.

The Society then adjourned.

*525 North Howard Street.* DR. H. O. REIK, *Secretary.*

